# Discovery of Novel New Delhi Metallo-β-Lactamases-1 Inhibitors by Multistep Virtual Screening

**DOI:** 10.1371/journal.pone.0118290

**Published:** 2015-03-03

**Authors:** Xuequan Wang, Meiling Lu, Yang Shi, Yu Ou, Xiaodong Cheng

**Affiliations:** 1 School of Life Science and Technology, China Pharmaceutical University, Nanjing, People’s Republic of China; 2 Department of Integrative Biology & Pharmacology, The University of Texas Health Science Center, Houston, United States of America; University of British Columbia, CANADA

## Abstract

The emergence of NDM-1 containing multi-antibiotic resistant "Superbugs" necessitates the needs of developing of novel NDM-1inhibitors. In this study, we report the discovery of novel NDM-1 inhibitors by multi-step virtual screening. From a 2,800,000 virtual drug-like compound library selected from the ZINC database, we generated a focused NDM-1 inhibitor library containing 298 compounds of which 44 chemical compounds were purchased and evaluated experimentally for their ability to inhibit NDM-1 *in vitro*. Three novel NDM-1 inhibitors with micromolar IC_50_ values were validated. The most potent inhibitor, VNI-41, inhibited NDM-1 with an IC_50_ of 29.6 ± 1.3 μM. Molecular dynamic simulation revealed that VNI-41 interacted extensively with the active site. In particular, the sulfonamide group of VNI-41 interacts directly with the metal ion Zn1 that is critical for the catalysis. These results demonstrate the feasibility of applying virtual screening methodologies in identifying novel inhibitors for NDM-1, a metallo-β-lactamase with a malleable active site and provide a mechanism base for rational design of NDM-1 inhibitors using sulfonamide as a functional scaffold.

## Introduction

Antibiotics being used to treat or prevent infectious disease have revolutionized the practice of medicine. Without them numerous modern therapies such as organ transplantation and cancer chemotherapy would simply not be possible [1]. Unfortunately, overuse and/or misuse of antibiotics in farming and clinical practices has resulted in rising multidrug-resistance bacterial strains, among which gram-negative bacteria producing β-lactamases become the most prevalent [2–4]. According to the U.S. Centers for Disease Control and Prevention, more than two million people are affected by infectious diseases with antibiotic resistance and at least 23,000 people died each year in the United States [5].

β-lactamases have been classified into four classes (A-D) based on their structures (Ambler classification) [6–8], among which the class B enzymes also known as metallo-β-lactamases (MBLs) requiring bivalent metal cation, normally Zn^2+^, as cofactors are further classified into B1, B2, and B3 subclasses [9]. B1 and B3 MBLs containing two zinc binding sites exhibit a broad substrate spectrum profile including the last antibiotic defense lines carbapenems, therefore pose a looming pandemic threat [10]. One typical example is the global dissemination of bacteria harboring B1 subgroup member New Delhi metallo-β-lactamase (NDM-1). These bacteria often carry several different resistance genes in addition to NDM-1 gene, *bla*_*NDM-1*_, and are resistant to almost all antibiotics and only partially susceptible to colistin, tigecycline, and fosfomycin, creating enormous challenges in managing these multi-resistance “Superbugs” [11–13]. In addition, colistin and tigecycline resistant NDM-1 harboring bacteria have been reported [14–16] and NDM-1 gene has been isolated in more than 11 bacterial species from natural environment [17–19]. Given the biomedical importance role of MBLs, development of MBLs inhibitors become an urgent need. High throughput screening (HTS) and virtual screening (VS) are the two main methods to identify novel scaffolds for drug discovery. Indeed, HTS has successfully identified a number of MBLs inhibitors [20–22], yet structure-based drug design and virtual screening have not been widely applied in MBLs inhibitors development [23]. Since the force field and zinc parameter has been optimized for metalloenzymes, molecular docking has proved to be a feasible way to found inhibitors or predict actual substrates of metalloenzymes structures [24–28]. Five inhibitors of CcrA, a B1 subclass of MBLs, with apparent Ki values less than 120 μM have been screened based on virtual screen method [29]. Recent high-resolution x-ray crystallographic analyses of multiple three-dimensional structures of NDM-1 reveal that it shares a common structural-fold with other B1 MBLs [30–34]. In addition, all three subclasses of MBLs share a common substrate hydrolysis mechanism [31]. These findings suggest that discovery of NDM-1 inhibitors via structure-based design and in silica screening may be productive. The aim of this study is to identify novel inhibitors of NDM-1 using virtual screening methods.

## Materials and Methods

### Bacterial strains and plasmids

MBL DNA sequences of NDM-1, VIM-2 and SIM-1 lacking the signal sequences were codon-optimized for expression in *E. coli*, chemically synthesized and inserted into pUC-19. Sequencing-validated MBL genes were further cloned into the pET28a expression vector using the NcoI/XhoI sites. *E. coli* DH5α (ATCC 53868) was used routinely as host for molecular cloning and plasmids amplifying while *E. coli* BL21 (DE3) was used for MBLs expression. Bacteria were grown in Luria–Bertani (LB) medium supplemented with appropriate antibiotics.

### Protein expression and purification

Recombinant NDM-1, VIM-2 and SIM-1 proteins were induced to express in *E. coli* BL21 (DE3) cells by 0.1 mM IPTG for 10 h at 2°C when the optical density (OD_600_ nm) reached 0.7–0.8. Cells were harvested and cell lysate was prepared by sonication at 4°C. The protein expression levels in soluble and insoluble fractions were analyzed by 12% SDS-PAGE after ultracentrifugation. Individual MBL was purified from the lysate supernatant using Ni^2+^-affinity column (Bio Basic Inc, Markham, Canada). All three recombinant proteins showed an abundant expression after induction for 10 h and could be purified with an estimated purity around 95% **(Figure A in S1 File)**. MBL activity analysis was carried out using the nitrocefin assay at 30°C in 300 μL HEPES buffer (30 mM HEPES, 10 μM ZnCl_2_, 100 mM NaCl, 20 μg/mL BSA, pH 6.8) at 482 nm with a UV-2400PC spectrophotometer (Shimadzu, Tokyo, Japan). The Michaelis constants, determined under initial velocity conditions by Lineweaver-Burk plot, for NDM-1, Vim-2 and SIM-1 were 9.54 ± 0.43 μM, 14.48 ± 0.68 μM and 31.3 ± 0.24 μM, respectively. These values are consistent with those previously reported [33, 35].

### Selection and preparation of structure models

22 reported NDM-1 X-ray crystallographic structures were analyzed [30–34,36] **(Table A in S1 File)** using protein alignment and superpose biopolymer module in Molecular Operating Environment suit (MOE, version 2009.10; Chemical Computing Group Inc; Montreal, QC, Canada) or the Protein Model Portal (PMP) [37] to facilitate the structure-based virtual screening. Structure 3Q6X (**Figure B.A in S1 File**) with a resolution value of 1.30 Å was selected for the screening process. The structural file contains two almost identical NDM-1 molecules with an RMSD value of 0.21 Å for C_α_ atoms [31]. The second structure after removing ligands and non-conserved water molecules in the active site, was processed for Protonate 3D and Energy Minimize using MOE. All hydrogen atomic coordinates were refined by the conjugate gradient method using the MMFF94x (Merck Molecular Force Field 94x) force field [38]. Other 21 NDM-1 structures were also processed with ligand and solvent deletion, protonate 3D and energy minimization using the same parameters and superposed together.

### Initial virtual screening

Hydrolyzed ampicillin, L-captopri, ampicillin and other 9 β-lactams (cefepime, cefotaxime, ceftazidime, cefuroxime, faropenem, imipenem, meropenem, penicillin G, piperacillin) structures downloaded from ZINC database were docked into the NDM-1 active site using different docking simulations in MOE and docking protocols in Discovery Studio (ADS, version 2.5; Accelrys Inc, San Diego, USA) according to the following procedure: the docking box was generated around the active site using the site finder module in MOE (**Figure B.B in S1 File**). The dimensions of the docking box were manipulated to accommodate all the amino acid residues present in the active site. Default parameters were used for all computational procedures unless otherwise stated.

A virtual collection drug-like compounds subset taken from ZINC database containing 2,800,000 compounds was served as the screening library [39]. The hits with firm binding conformations were collected and redocked into the active site using the libdock protocol in ADS. Those compounds with high libdock scores were selected as a focused library used for the further analysis.

### Docking results analysis

Energy calculations and analysis of docking poses were performed on MOE. The resulting protein-inhibitor or protein-β-lactam complexes were analyzed using the protein–ligand interaction fingerprint (PLIF) implemented in MOE [40]. The hydrolyzed ampicillin and NDM-1 residue interaction energies were calculated for the docked pose with the least RMSD value, assigning energy terms in kcal mol^-1^ for each residue. LigX-interaction application was used to provide ligand-interaction diagram to understand the binding type of those docked hits [41].

### A 96-well assay for NDM-1 inhibitor screening

Preliminary screening of the selected compounds was performed in 96-well plates using nitrocefin as a substrate. Final assay conditions include compounds (30 μM), NDM-1 (1 nM), HEPES (30 mM), ZnCl_2_ (10 μM), NaCl (100 mM), BSA (20 μg/mL) at pH 6.8. EDTA (30 μM) was used as a positive control. After incubation at 30°C for 20 min, nitrocefin hydrolysis (100 μM) was monitored by following absorbance readings at 490 nm using a PR 4100 Microplate Reader (BIO-RAD; USA). The assay was performed in quadruplicate for all compounds and controls.

### IC_50_ Determination

Ten different concentrations of compounds VNI-24, VNI-34 and VNI-41 ranging from 0 μM to 45.0 μM were used to determine the half-maximal inhibitory concentration (IC_50_) against NDM-1 (1 nM) using nitrocefin (20 μM) as substrate. The assay was performed in the buffer for inhibitor screening in the presence or absence of 0.01% Triton X-100 [42]. Each data point was performed in quadruplicate and the inhibition data were analyzed by a standard dose response curve fitting in the Origin 8.0 software.

### Analysis of NDM-1/VNI-41 complexes by molecular dynamics study

NDM-1 and VNI-41 after optimized with partial charge were then subjected to molecular dynamics simulation (MD) employing the NVT (N = constant number, V = volume, and T = temperature) statistical ensemble and Nosé-Poincaré-Andersen (NPA) algorithm with the periodic boundary conditions applied to analysis stability of binding model of the compound. The complex was solvated in water molecules in a sphere mode with a 10 Å width layer. The molecular dynamics simulations were performed at a temperature of 310 K for 2000 ps. The data of position, velocity and acceleration were saved every 0.5 ps.

## Results and Discussion

### NDM-1 structural superposition and optimization

**Fig 1:**
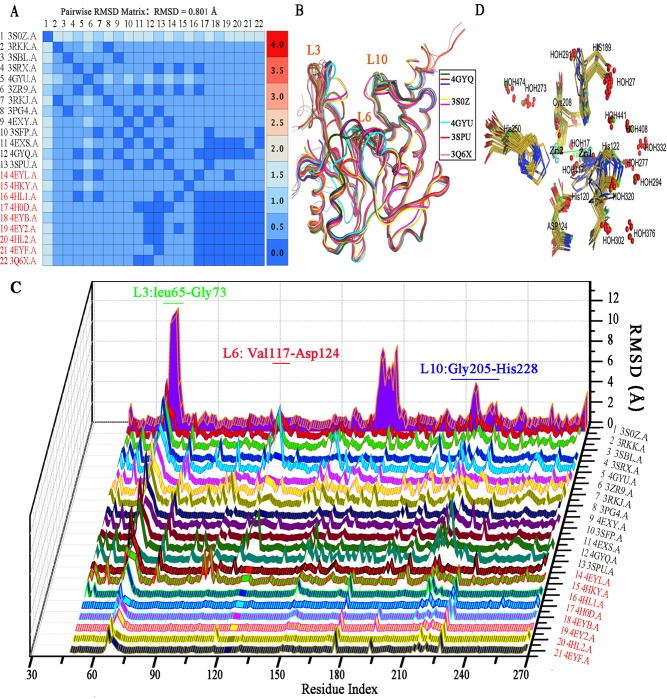
Comparative analyses of 21 published NDM-1 X-ray crystal structures. (A) Pairwise RMSD matrix table of NDM-1 structures superimposed with force realignment method and refine with Gaussian Weights in MOE. PDB codes for structures with hydrolyzed substrate in the active site are highlighted in red. (B) Superposition of the 22 NDM-1 structures. 3S0Z, 4GYU, 4GYQ, 3SPU, and 3Q6X2 are highlighted in thick line and colored as shown in the index panel. (C) The RMSD-residue index 3D waterfall plots of NDM-1 structures compared with 3Q6X structure. (D) Superimposition of the active site among the reported NDM-1 structures (without 3S0Z and NDM-1 mutants 4GYQ and 4GYU) showing the metal chelating residues (Oliver) and conserved water molecules (Red) in the active site of NDM-1 structures. Residues from 3Q6X are highlighted in green.

Structural superposition of the 22 reported NDM-1 structures using force realignment and refined with gaussian distance weight showed that most of the independently solved structures (except 3S0Z) shared a high degree of structural similarity with each other (**Figure 1A, 1B, Figure C in S1 File**). The average pair-wised RSMD for all atoms in these structures is 0.801 Å (**Fig. 1A**) and 3Q6X showed a high similarity with 4EYF, 4HOD, 4HL1, 4HL2, 4EYB and 4EY2 with RMSD values below 0.3 Å (**Fig. 1A, Table A in S1 File**). A notable variation is the distance between zinc ions ranging on average from 3.48 to 4.6 Å **(Table A in S1 File, Figure B.C in S1 File)**. This indicates that the metal ions are relative flexible to move within the active site. While NDM-1 structures complexed with hydrolyzed antibiotics share a greater metal-ion separation (4.53 ± 0.11Å, 3Q6X 4.59 Å) with a slight outward flexing of His120 and His122, the binuclear Zn distance appears to be significantly less (3.72 ± 0.17Å, p < 0.001) in most apo-NDM-1 structures **(Table A in S1 File)**. Since the binuclear Zn distance is compatible with μ-η1:η1carboxylate coordination, such distance changes also prevail in all other binuclear Zn MBLs [43], and this inherent flexibility of metal ions in the active site is likely important for substrate binding and turnover [36,44]. 4H0D and 4HL1, where the Zn ions replaced by Mn^2+^ or Cd^2+^ showed a similar hydrolyzed ampicillin binding framework as 3Q6X [45]. A detailed RMSD analysis for 3Q6X reveals that residues in loop L3 (Leu65-Gly73) adjacent to the active site possess greatest variation **(Fig. 1C).** Moreover, L3 displayed the greatest deviations with the exception of the N terminal signal peptide when structures with hydrolyzed antibiotics (4HKY, 4EY2, 4EYF, 4HL1, 4HL2, 4H0D. 4EYB and 3Q6X) were superposed with apo structures while L3 showed less difference among 3Q6X, 4HL2 (Hydrolyzed Ampicillin), 4EYB (Hydrolyzed Oxacillin), 4EYF (Hydrolyzed Benzylpenicillin) and 4EY2 (Hydrolyzed Methicillin) (**Figure C.C & C.D in S1 File**). These results suggest that L3 is involved in substrate binding in NDM-1. Loop L10 (Gly205-His228) showed the subordinate deviation after L3 among NDM-1 structures (**Fig. 1C, Figure C.C in S1 File**). Asn220 in L10 interacts with Zn1 to provide an oxyanion hole in polarizing the lactam carbonyl upon binding, and facilitates nucleophilic attack by the adjacent hydroxide [32]. Regions of Ala121–Met129 flanking NDM-1 active site also showed a slightly difference among NDM-1 structures especially when NDM-1 mutants 4GYQ (D223A) and 4GYU (A121F) superposed with 3Q6X (**Figure C.D in S1 File**). A detailed analysis showed that the loop spanning residues Phe163–Asn176 in 3S0Z adopt a very different conformation from those observed in the other structures, and the so called “ceiling” region in 3S0Z is a loop while in most other reported structures it is an α-helix (**Figure C.F in S1 File**). Quantitative stability-flexibility relationship analysis revealed that NDM-1 had several regions with significantly increased rigidity when compared with other four B1 MBLs [46]. In most NDM-1 structures (except mutants and 3S0Z), RMSD of these regions were blow 0.5 Å **(Figure C.A in S1 File)**. These evolutionary traits of NDM-1, with more rigid regions out of the active site together with the more plastic and more hydrophobic L3 loop [31] as compared to other MBLs, may provide more flexibility to accommodate a broader spectrum of substrates.

Based on our detailed analysis, many NDM-1 structures shares some identical waters in the active site (**Fig. 1D**), which may play a role in the overall structure stability or in substrate binding and product turnover. The bridging water in the active site showed different distances among these structures, a likely consequence of change in distance between the metal ions (**Figure B.D in S1 File**) or the pH conditions that the protein crystallization were used. Unlike most other MBLs, NDM-1 functions well at high pH conditions [47]. Our analyses suggest that 3Q6X is of high resolution and possesses a high degree of the structural similarities with other NDM-1 structures, therefore is suitable for docking and screening studies.

### Molecular docking

**Fig 2:**
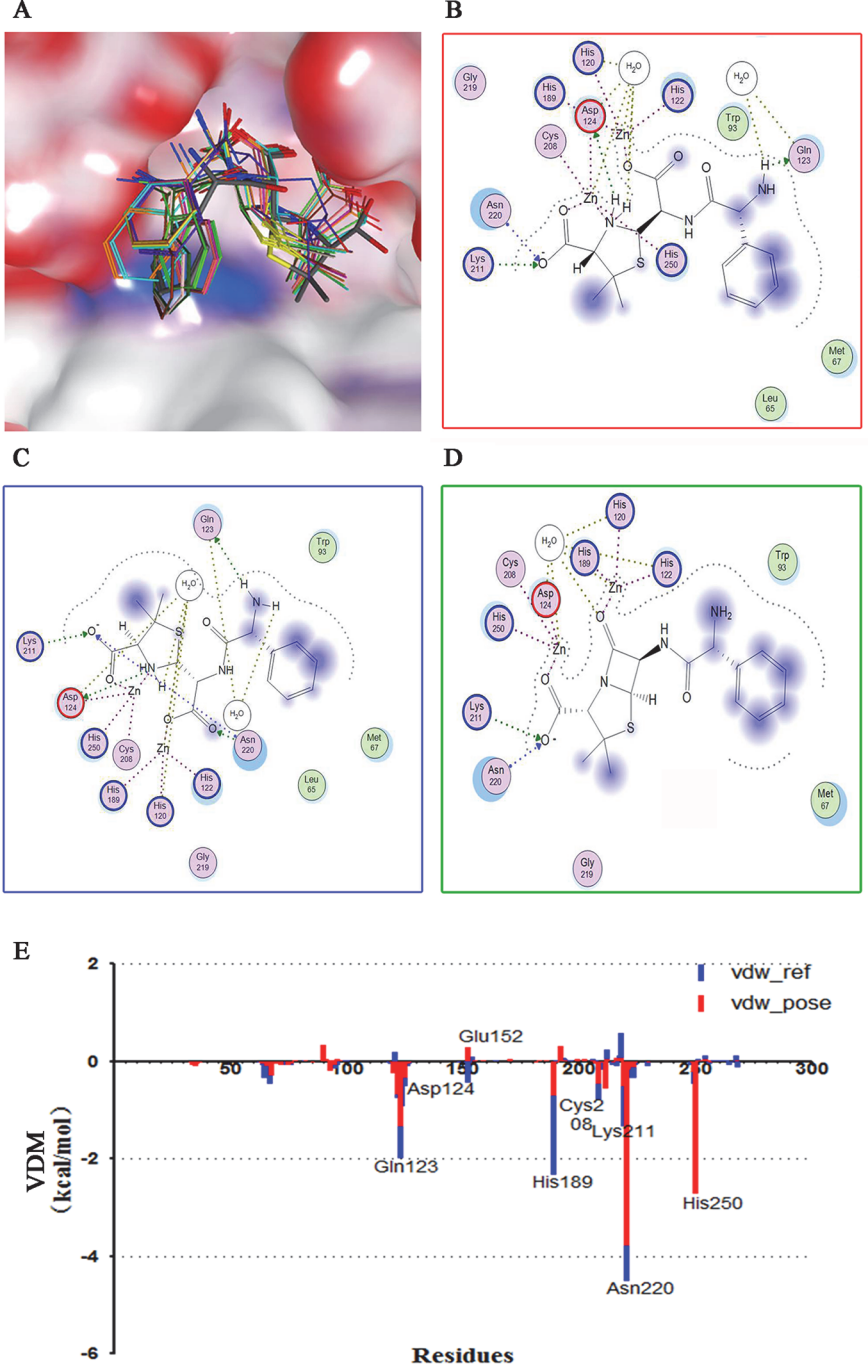
Docking of the hydrolyzed ampicillin in the active site of NDM-1. (A) Molecular surface of NDM-1 (PDB 3Q6X) active site with docked hydrolyzed ampicillin. The structurally determined hydrolyzed ampicillin is shown in gray stick representation while docked poses are shown in colored stick. 2D ligand-protein interaction maps showing the detailed binding pattern of structurally determined hydrolyzed ampicillin (B), docked hydrolyzed ampicillin (C) and docked ampicillin (D) in the active site of 3Q6X. (E) Residue-ligand interaction energies between NDM-1 (3Q6X) and hydolyzed ampicillin (vdw_ref) or docked hydolyzed ampicillin (vdw_pose). The hydrolyzed ampicillin and NDM-1 residue interaction energies were calculated for the best pose (RMSD = 1.53 Å).

**Fig 3:**
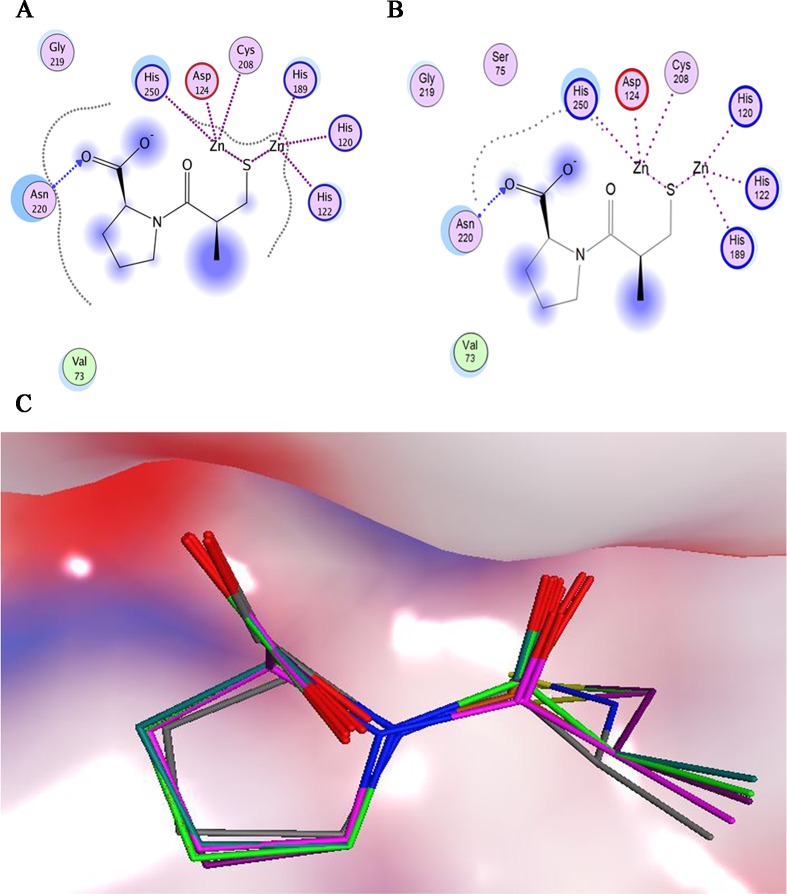
L-captopril docked in the active site of NDM-1. 2D ligand-protein interaction maps showing the detailed binding pattern analysis of structurally determined (A) and docked (B) L-captopril. (C) Molecular surface of NDM-1 active site with docked hydrolyzed ampicillin.

**Fig 4:**
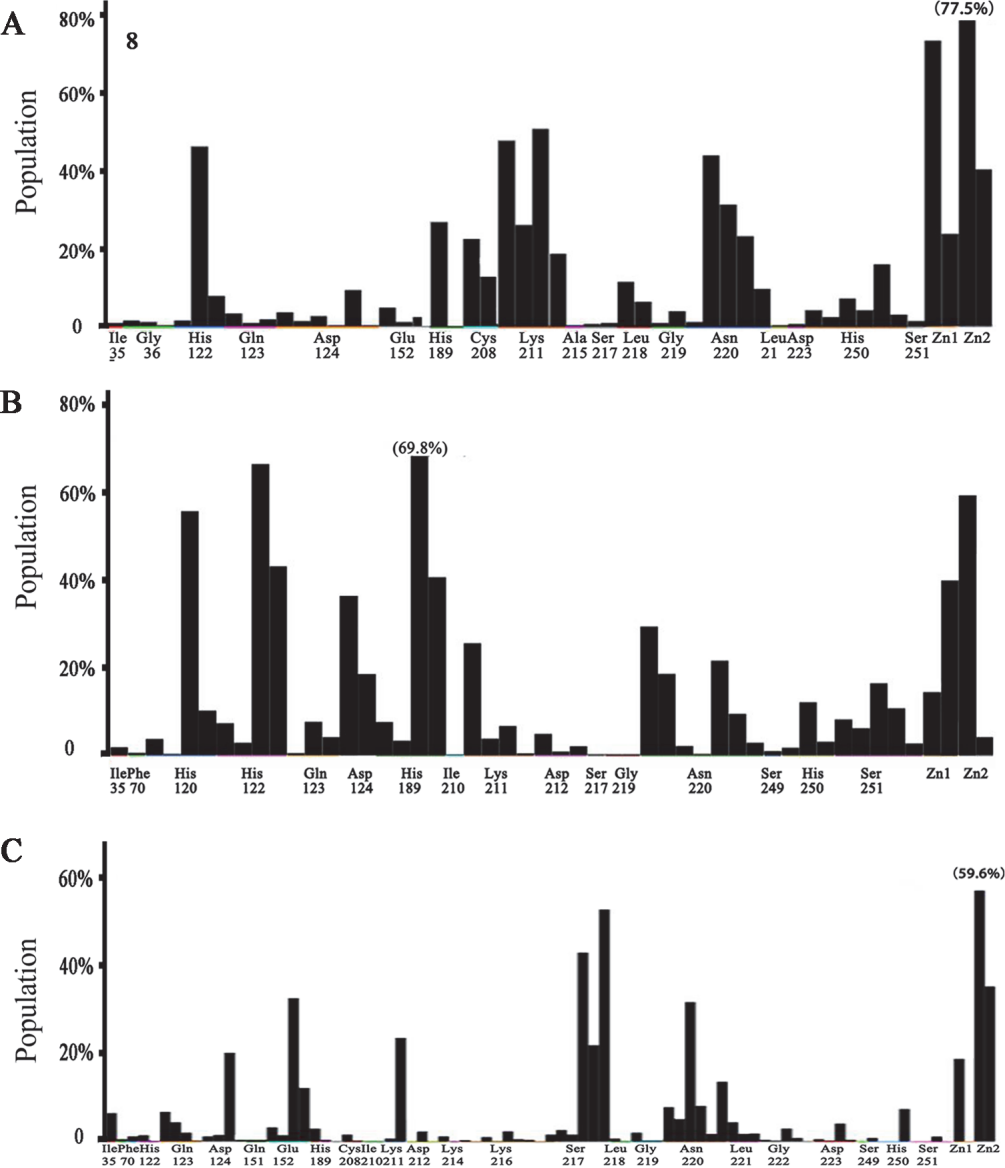
PLIF analysis of the docking process. The interaction frequency of individual residue with the docking poses of (A) hydolyzed ampicillin; (B) 10 beta-lactams (ampicillin, cefepime, cefotaxime, ceftazidime, cefuroxime, faropenem, imipenem, meropenem, penicillin G, piperacillin); (C) 298 virtual hit compounds. Each columns of every residue are denoted by some of the following characters to indicate the interaction role of each residue: side chain hydrogen bond acceptor, backbone hydrogen bond donor, backbone hydrogen bond acceptor, solvent hydrogen bond, ionic attraction or surface contact to the atom of the residues.

Hydrolyzed ampicillin, L-captoril, ampicillin and other 9 β-lactams (cefepime, cefotaxime, ceftazidime, cefuroxime, faropenem, imipenem, meropenem, penicillin G, piperacillin) were docked into NDM-1 active site using different docking simulations in MOE and docking protocols in ADS to evaluate the ability of these programs to reproduce the experimental binding modes. For all programs the binding modes of the docked hydrolyzed ampicillin structures were found in a narrow range of RMSDs (**Fig. 2A**). The RMSDs of hydrolyzed ampicillin were 1.53–2.07 Å, 1.86–2.62 Å, 1.98–2.78 Å and 1.79–2.31 for Triangle Matcher, Alpha PMI, Alpha triangle, Proxy triangle placement in MOE respectively, while 1.46–2.65 Å for libdock in ADS receptor-ligand interactions protocols. In general, poses with an RMSD < 2 Å are considered a success, and dockings with RMSDs between 2 and 3 Å are considered a partial success [48]. For L-captopril, the RMSD values between poses docked into the active sites and the determined ligand structure in 4EXS arranged from 0.72 to 2.03 Å and the 2D interaction map of the docked L-captopril was similar to that in 4EXS (Fig. 3). Hydrolyzed ampicillin-residue interaction energies for the best docked pose and the structure reference were calculated [49]. The interaction of best docked hydrolyzed ampicillin and ampicillin showed similar interactions as revealed by the X-ray structure (**Fig. 2B, 2C, 2D**). Among the conserved residues in the active site, Leu65, Gln123, Asp124, His189, Cys208, Lys211, Asn220 interact with the hydrolyzed ampicillin in both the structure complex and the docked pose. PLIF analysis showed that Gln123, His189 and Asn220 interacted with the docked hydrolyzed ampicillin at a high frequency **(Fig. 4A**). The residue interaction energies between NDM-1 and hydrolyzed ampicillin, and the 2D interaction map **(Fig. 2E)** well defined the dock results.

After ampicillin and other β-lactams were docked into the active site, the 2D binding pattern of these binding pose were analyzed by the ligX-interactions and RLIF. 404 docked poses of 10 different β-lactams showed that those substrates interacted with His120, His122, Asp124, His189, Lys211, Ser249, Asn220, Zn1 and Zn2 at a high frequency compared with other residues around the docking site. On the other hand, other residues Ile35, Phe70, Asp212 and Ser217 interacted less frequently (**Fig. 4B**). Docking poses also formed the inhibited conformers at a low frequency (< 10%), in which the carboxylic group of β-lactams coordinated with two zinc ions and kept the amide group away from the metal ions as described before [50]. These findings, along with the high flexibility of NDM-1 active site, is consistent with NDM-1’s broad substrate spectrum, as well as the fact that most reported MBL inhibitors interact with Zn^2+^ or Zn^2+^ chelating residues [51].

### Structure-based screening and analysis

Triangle Matcher placement method, followed by molecular mechanics refinement and scoring, was used for the first round docking based screening process. The placement stage was scored by E_place. Binding free energy values (G_binding_) was quantified using London dG [52] and Affinity dG [53]. After 1000 binding orientations for each compounds were refined, 30 conformations with lowest binding free energy, lowest affinity dG and London dG values was produced. In the screening process, we adopted the strategy that the most anticipated hits would exhibit the desirable scores in all the evaluation algorithms and be in conformity with screening threshold of different screening methods.

Docking poses without major clashes were scored for receptor complementarity and were further screened using the criteria that affinity dG value was less than -10 kcal/mol and that london dG was less than -20 kcal/mol. E_refine for refinements using GridMIn was limited to 190 kca1/mol. E_conf, the energy of the conformer calculated at the end of the refinement was confined to less than 9 kcal/mol **(Figure C.D in S1 File)**. 2218 compounds satisfied these filter settings were selected from the database.

In the second round of screening, the receptor-ligand interactions protocols of ADS 2.5 were used to dock these 2218 compounds into the docking box of NDM-1. For each ligand, another 30 different conformations for each compound were generated by the libdock process. On the basis of the docking scores, the compounds were ranked, and 1388 conformations of 298 compounds with libdock score above 150, Absolute Energy under 200 kcal/mol; Relative Energy under 25 kcal/mol were selected.

The 1388 screened conformations displaying in a camel-like appearance (**Figure D.A, D.B & D.C in S1 File**) were further analyzed by PLIF. During the PLIF screening, we focused on interactions with His122, His189, Asn220, His250, Zn1, and Zn2 because all these elements showed high interaction frequency in the β-lactam based docking (**Fig. 4B**). 1,388 conformations (poses) of 298 compounds satisfied with above specific binding requirement were selected as a focused library, and most of which also interact with Ile35, Gln123, Asp124, Lys211, Ser217, Gly219 and Ser251 at a high frequency **(Fig. 4C)**. In addition, these molecules were inspected visually for features not captured in the docking calculation.

### Biological activity analysis of the screened compounds *in vitro*

**Fig 5:**
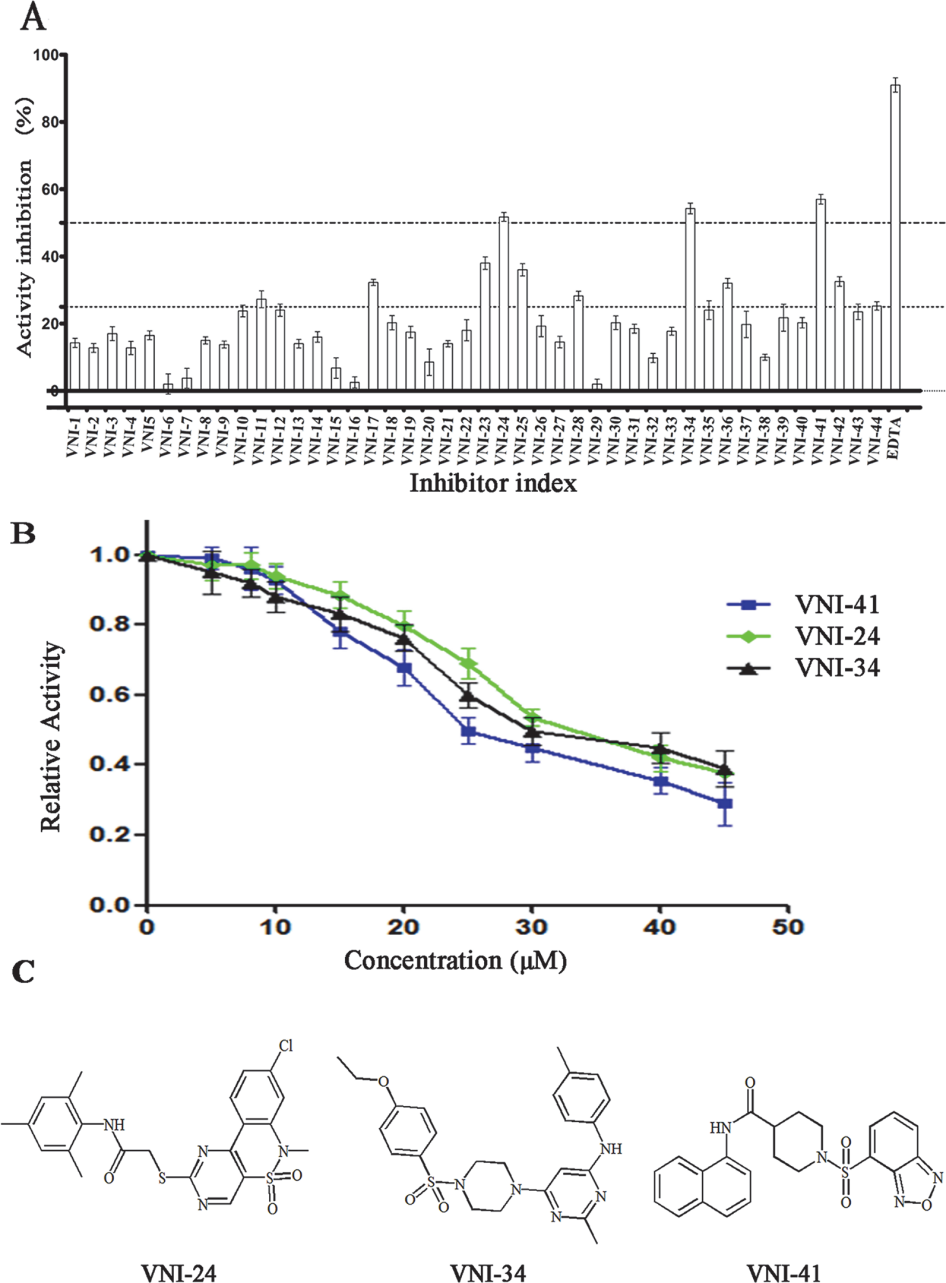
Experimental validation of selected virtual screening hits. (A) Percent inhibition of NDM-1 activity in the presence of 30 uM individual compounds. Data are presented as mean ± standard deviation (n = 4). (B) Dose-dependent inhibitions of NDM-1 by VNI-24, VNI-34 and VNI-41 against NDM-1. Each data point indicated the remaining activity of NDM-1 after incubated with inhibitors, and were presented as mean ± standard deviation (n = 4). (C) Structures of three active compounds.

Based on the docking scores, chemical diversity, 2D ligX-interactions map, commercial availability, and an overall balance between polar and nonpolar complementarity to the binding site, 44 molecules (**Table B in S1 File**) were ultimately selected and purchased from the ChemDiv (San Diego, CA) for experimental validation using *in vitro* assays. When the selected 44 chemicals were tested for their ability to inhibit NDM-1 activity, eleven compounds showed more than 25% inhibition at 30 μM concentration. Among these eleven compounds VNI-24, VNI-34, and VNI-41 inhibited NDM-1 by more than 50% at 53.2% ± 2.2%, 56.5% ± 2.6%, and 56.8% ± 3.0%, respectively (**Fig. 5A**). Dose-dependent analyses further revealed that compounds VNI-41, VNI-34 and VNI-24 inhibited NDM-1 with an apparent IC_50_ value of 29.6 ± 1.3 μM, 31.4 ± 1.2 μM, or 37.6 ± 0.9 μM, respectively **(Fig. 5B, 5C).** Similar results were obtained using buffer containing 0.01% Triton X100 (**Figure E in S1 File**).

Activity of the three compounds against VIM-2 and SIM-1 was also tested. Within the aqueous solubility limit of these compounds (**Table C in S1 File)**, none of the three compounds showed significant inhibition for SIM-1. While 45 μM VNI-24 and VNI-41 inhibited VIM-2 activity by 19.6% ± 3.1% and 34.2% ± 5.2%, respectively, VNI-34 was ineffective in blocking VIM-2 activity. These results suggest that VNI-24, VNI-34 and VNI-41 are selective NDM-1 inhibitors capable of discriminating among various MBLs. Taken together, our study shows that it is feasible to develop novel NDM-1 specific inhibitors via *in silico* screening.

### Molecular dynamic study of the NDM-1/VNI-41 complex

**Fig 6:**
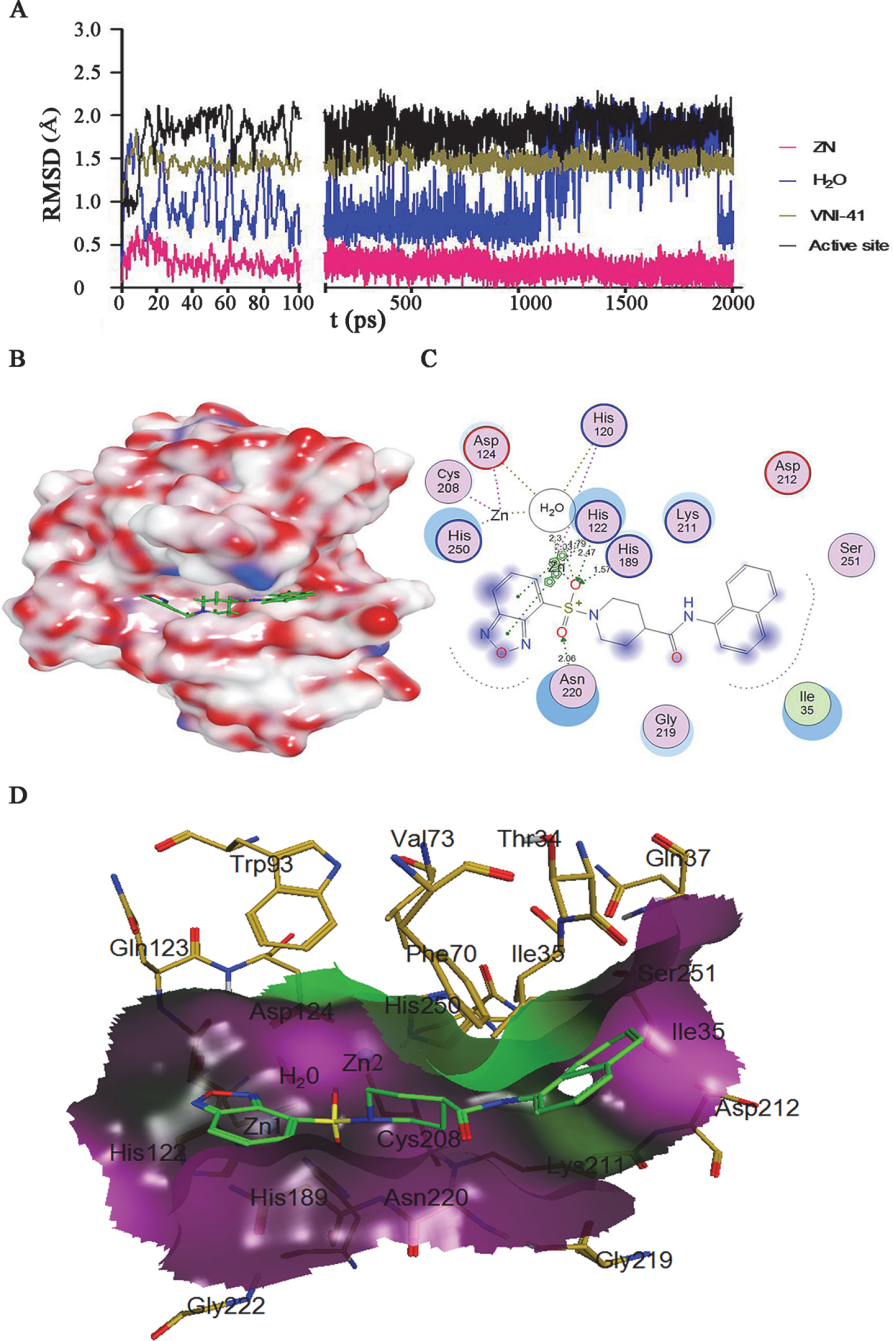
Molecular dynamic profile of NDM-1 and VNI-41 complex. (A) RMSD of two Zn^2+^, conserved H_2_O, VNI-41, and active site (atoms shown in Fig. 1D) spanning the 2000 ps molecular dynamic simulation process; (B) The overall molecular surface of NDM-1 colored by activeLP (white, hydrophobic; blue, polar; red, H-Bonding); (C) The 2D interaction map VNI-41 in the active site of NDM-1 at 1500 ps after the MS reached equilibrium; (D) Molecular surface of NDM-1 around the VNI-41 binding site with a cutoff limit of 4.5 Å (black, hydrophilic; purple, neutral; green, lipophilic). VNI-41 and adjacent NDM-1 residues shown in stick representation with carbon atoms colored by green and dark yellow respectively.

**Fig 7:**
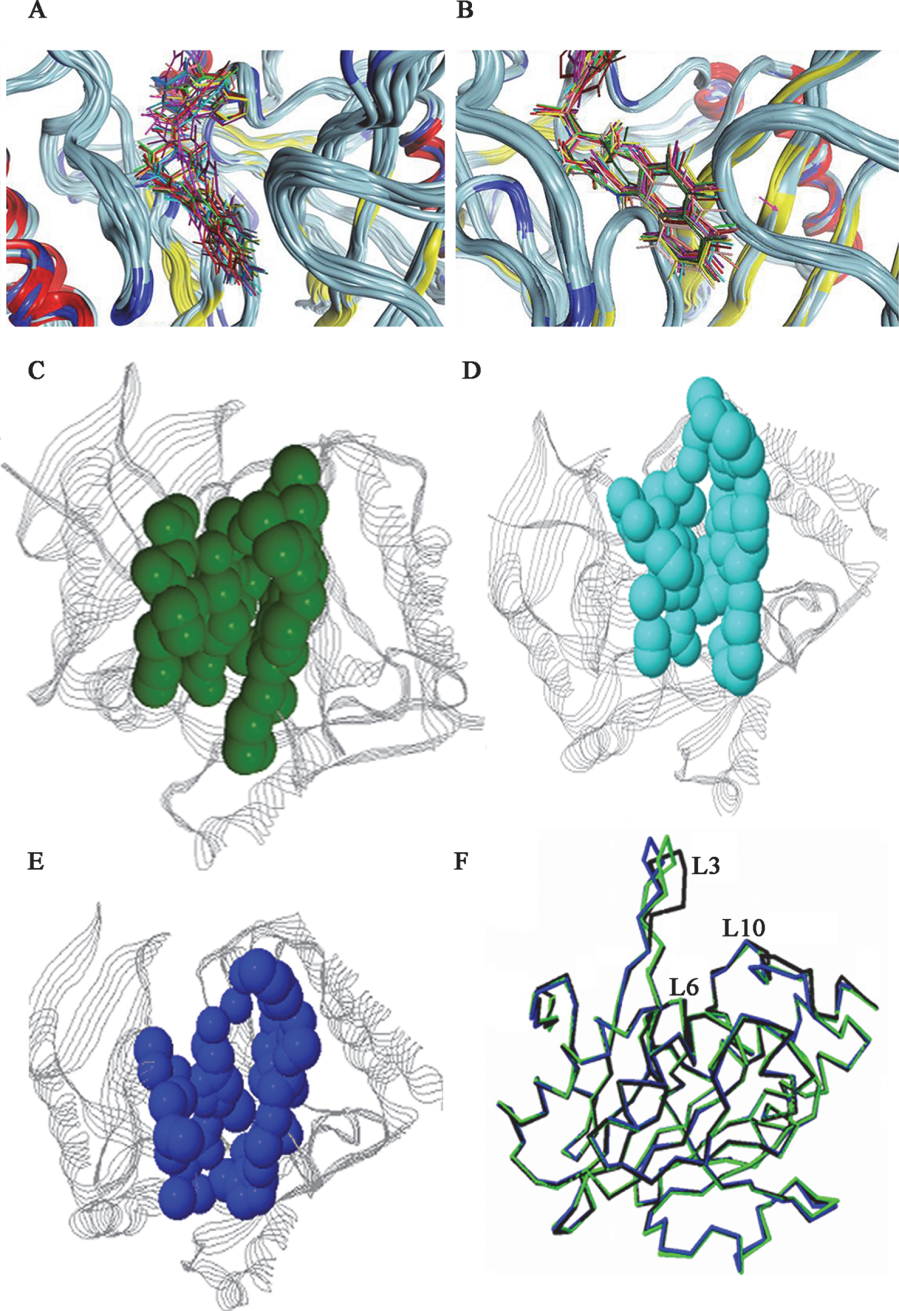
Structure movements during molecular dynamic simulation process. Overlaid snapshots of the ribbon diagrams of NDM-1 Cα atoms and VNI-41 compound around the active site before (A) (snapshots interval 10 ps, N = 10) and after (B) (snapshots interval 150 ps, N = 10) the system reached equilibrium. Surface analyse of the active site cavity was performed on apo NDM-1(C), NDM-1/hydrolyzed ampicillin (D) and NDM-1/NVI-41(E). Atoms in the active site active site cavity are highlighted in colored balls. (F) The ribbon diagrams showing the active site associated loops (L3, L6, L10) moving toward the ligand and contraction of the active site. The structure of apo NDM-1, NDM-1/hydrolyzed ampicillin and NDM-1/NVI-41 is colored in blue, green and black respectively.

To investigate stability of the active site cavity in response to the binding of VNI-41, the most potent NDM-1 inhibitor validated in our study, MD simulations were performed. RMSD for zinc ions, VNI-41 and the active site atoms (atoms in **Fig. 1D**) of NDM-1 from their initial positions (t = 0) was calculated. Overall, the RMSD values of NDM-1 active site fluctuated from 0.5 to 2.5 Å and reached a steady state **(Fig. 6A)** that the systems were equilibrated and the predicted pose of each inhibitor was compatible with the pocket in the catalytic cavity of NDM-1 structure. Close examination of MD simulation snapshots (N = 10, with different time intervals) of the VNI-41/NDM-1 complex relative to the original pose revealed a coordinated movement of L3, L10 and L12 around the active site **(Fig. 7A, 7B)**. The distance of the zinc ions maintained a steady state during the dynamic simulation and the RMSD of the zinc was less than 0.5 Å. While the ligand underwent a maximal change with a RMSD value of 1.6 Å, the active site showed a change with a RMSD value about 2.0 Å. The conservative water in the active site fluctuated and this may be caused by the solvent used to dissolve NDM-1 in the MD simulation competing with the water kept before MD simulation **(Fig. 6A**).

It is reported that the expanded cavity volume of the active site in the surrounding loops (Loop L1, L3, L10 and L12 in **Figure B.A in S1 File**) is important for the broad substrate spectrum of NDM-1 [33]. To investigate residue movement in the active site cavity in response to VNI-41 binding, a surface analysis was performed on apo NDM-1 and NDM-1/NVI-41 complex after MD simulation[54]. Apo NDM-1 has the largest cavity (surface area = 392.4 Å^2^; volume = 693.2 Å^3^) while NDM-1-hydrolyzed ampicillin complex has a smaller cavity after removing the ligand (surface area = 376.2 Å^2^; volume = 633.5 Å^3^). Active site cavity of NDM-1-VNI-41 complex is the smallest after removing the docked ligand (surface area = 345.3 Å^2^; volume = 596.3 Å^3^) (Fig. 7C, 7D, 7E). Our study shows that VNI-41 clamped into the groove surrounded by active site, induced L3 and L10 movement and narrowed the active cavity **(Fig. 7F)**.

Interactions between NDM-1 and compound VNI-41 among the MD generated steady conformations during MD simulation were analyzed. The benzoxadiazole moiety binding to NDM-1 hydrophilic site adopted an appropriate conformation with the double π-π stacking interactions with His122, and the ring-to-ring distances were 3.03 and 2.79 Å for the five member ring and the six number ring interacted with His122, respectively (**Fig. 6B, Fig. 6C**). Moreover, one oxygen atom from the sulfonamide group interacted with Zn2 via a metal contact (score 100%, distance 2.5 Å), forming a solvent contact with the bridge water (H_2_O in the 2D interaction map) (score 33%, distance 3.0 Å), and ionic contacted with His122, His120 and His189 (score 61%, distance 1.9 Å; score 42%, distance 1.8 Å; score 25%, distance 1.8 Å). The other oxygen atom interacted with Asn220 (score 69%, distance 2.06 Å). The naphthalene group clamped into hydrophobic groove around the active site and contacted with His250 and Ile30 (**Fig. 6D**). Mechanisms study has showed that the bridging hydroxide-zinc serving as the general base while a surrounding water molecule serving as the nucleophile responsible for the nucleophilic attack which results in a negatively charged intermediate stabilized by oxyanion hole of NDM-1 [45]. In the conformation of VNI-41 interacting with NDM-1, the bridge water formed solvent contacting with oxygen atom from sulfonamide group may prevent the proton transfer from the surrounding water to the bridging water in the active site.

To date, various sulfamide/sulfonamide/sulfamate containing metalloenzyme inhibitors, such as diuretic and antiglaucoma agents (acetazolamide, methazolamide, dichlorophenamide, and brinzolamide), have been clinically used to inhibit carbonic anhydrases [55]. Sulfamide/sulfonamide/sulfamate containing MBL inhibitors have also been reported. The crystal structure of 4-nitrobenzenesulfonamide interacting with BJP-1, a B3 subclass MBLs, reveals that binding of sulfonamide changes coordination number and geometry for Zn1 by adding one oxygen atom of sulfonamides to the Zn2 and the nitrobenzene moiety form a hydrophobic pocket in the active site [56]. MBL inhibitor DansylCnSH can be docked and shown to interact with the core region of the active site of IMP-1 via sulfamide [57]. Since the zinc ions of NDM-1 are essential for catalytic activity and participate directly in the catalysis, the ability of the sulfonamide group of VNI-41 to interact with the Zn ion in the active site of NDM-1 suggests that sulfamide/sulfonamide/sulfamate containing compounds may represent promising leads for developing clinically effective NDM-1 inhibitors.

In summary, we have identified novel inhibitors of NDM-1 using a multistep docking methodology. Dynamic and ligX-interaction analyses have revealed that VNI-41 interacts with Zn1. This study has demonstrated the feasibility of identifying inhibitors of NDM-1 with a plastic active site by virtual screening. Further investigations and future modifications studies for rational design of NDM-1 inhibitors using sulfonamides as a functional scaffold will lead to a better understanding of their exact mechanism of action, laying a solid foundation for further structure-based hit-to-lead optimization.

## Supporting Information

S1 File Supporting Information. (PDF)
