# Supplementary material for: Discovery of Novel New Delhi Metallo-β-Lactamases-1 Inhibitors by Multistep Virtual Screening
Source: PLoS One. 2015 Mar 3;10(3):e0118290. doi: 10.1371/journal.pone.0118290 (PMC4348537; doi:10.1371/journal.pone.0118290)
Supplement: S1 File — (PDF) [file pone.0118290.s001.pdf]

Supporting Information

Discovery of Novel New Delhi Metallo-β-lactamases-1 Inhibitors by Multistep Virtual Screening

Xuequan Wang[1,‡], Meiling Lu[1,‡], Yang Shi, Yu Ou[1,*], Xiaodong Cheng[2,*]

1 School of Life Science and Technology, China Pharmaceutical University, Nanjing 210009, People's Republic of China

2 Department of Integrative Biology & Pharmacology, The University of Texas Health Science Center, Houston, United States of America

‡ These authors contributed equally.

* Correspondence: Yu Ou: ouyu2008@126.com; Xiaodong Cheng: xiaodong.cheng@uth.tmc.edu

Table A. Structural similarity between 3Q6X and other NDM-1 structures

PDB ID	RMSD (Å)	Resolution (Å)	Aligned Residues	Sequence Identity	Chains	Metal ions	Distance of metals (Å)	Details	Ref
**3Q6X**	0	1.30	243	1.00	2	Zn^{2+}（1,2）	4.58	Hydrolyzed Ampicillin	30
**4EYF**	0.16	1.80	241	1.00	2	Zn^{2+}（1,2）	4.63	Hydrolyzed Benzylpenicillin	29
**4HL2**	0.16	1.05	241	0.99	2	zn^{2+}（1,2）	4.6	Hydrolyzed Ampicillin	□
**4EY2**	0.17	1.17	241	1.00	2	Zn^{2+}（1,2）	4.58	Hydrolyzed Methicillin	29
**4EYB**	0.17	1.16	241	1.00	2	Zn^{2+}（1,2）	4.55	Hydrolyzed Oxacillin	29
**4H0D**	0.19	1.50	241	0.99	2	Mn^{2+}（1,2）	4.49	Hydrolyzed Ampicillin	□
**4HL1**	0.28	1.50	238	1.00	2	Cd^{2+} (1,2)	4.3	Hydrolyzed Ampicillin	□
**3SPU**	0.54	2.10	230	1.00	5	Zn^{2+}（1,2）	3.56	Apo Ndm-1 Structure	32
**4HKY**	0.56	2.0	228	1.00	2	Cd^{2+} (1,2)	3.48	Hydrolyzed Faropenem	□
**4GYQ**	0.61	1.35	229	0.99	3	△	—	D223A mutant	□
**4EXS**	0.61	2.40	229	1.00	2	Zn^{2+} (1,2)	3.66	L-Captopril	29
**3SFP**	0.62	2.27	229	1.00	4	ZN^{2+} (1,2)	—	delta-36NY construct, Mono- Zinc	32
**4EYL**	0.64	1.90	229	1.00	2	Zn^{2+}（1,2）	4.05	Hydrolyzed Meropenem	29
**4EXY**	0.65	1.47	229	1.00	2	Zn^{2+}（1,2）	3.63	Ethylene, Glycol	29
**3PG4**	0.69	2.0	223	0.99	1	△	—	No metal ions binding	□
**3RKJ**	0.8	2.0	223	1.00	2	△	—	delta-38 construct	32
**3ZR9**	0.83	1.91	230	0.99	1	Zn^{2+},Cd^{2+} (1, 2)	3.51	Apo Ndm-1 Structure	34
**4GYU**	0.84	1.80	222	1.00	1	△	3.66	A121F mutant	□
**3SRX**	0.85	2.50	230	1.00	2	Cd^{2+}(1,2)	3.64	Thiocyanatem ether, Glycerin	□
**3SBL**	0.87	2.31	228	1.00	3	△	—	Citric Acid	32
**3RKK**	0.91	2.35	225	1.00	2	△	—	Sulfate Ion, Glycerin, Acetic Acid	32
**3S0Z**	1.33	2.50	212	0.98	2	Zn^{2+}（1,2）	3.77	Apo Ndm-1 Structure	33

△: No metal binding in the active core; — only one or no metal ions in the active site. □ to be published.
RMSD: the root of the mean square distances between equivalent C-alpha atoms of all aligned residues between 3Q6X and other structure.

Table B. Compounds selected from the multistep docking virtual screening hits for experimental validation.

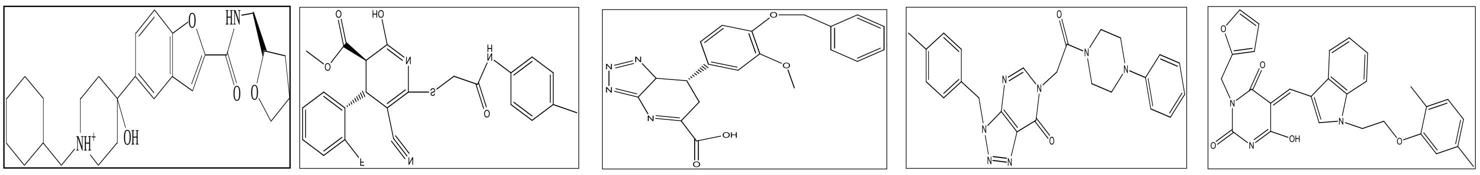

VNI-1 ZINC14957775 | VNI-2 ZINC01142185 | VNI-3 ZINC00942859 | VNI-4 ZINC09327463 | VNI-5 ZINC27548754

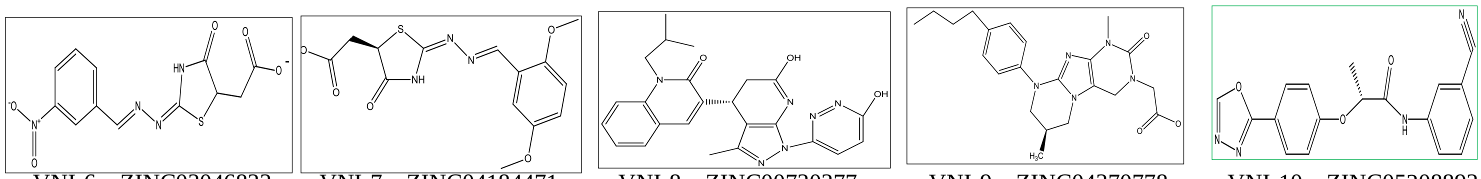

VNI-6 ZINC03046833 | VNI-7 ZINC04184471 | VNI-8 ZINC00730377 | VNI-9 ZINC04370778 | VNI-10 ZINC05208893

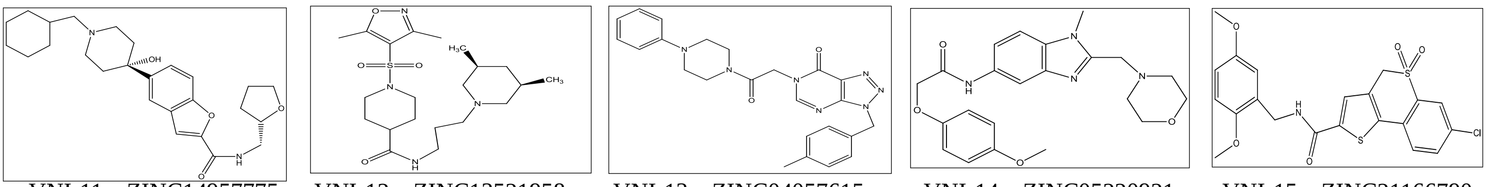

VNI-11 ZINC14957775 | VNI-12 ZINC13521958 | VNI-13 ZINC04057615 | VNI-14 ZINC05220921 | VNI-15 ZINC21166790

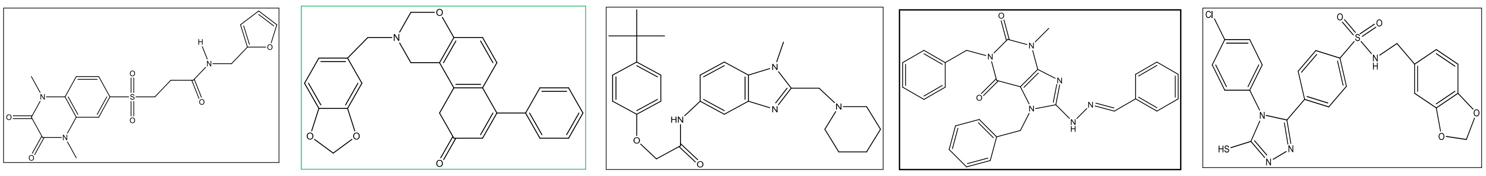

VNI-16 ZINC06808607 | VNI-17 ZINC20403810 | VNI-18 ZINC26120963 | VNI-19 ZINC26121611 | VNI-20 ZINC13992128

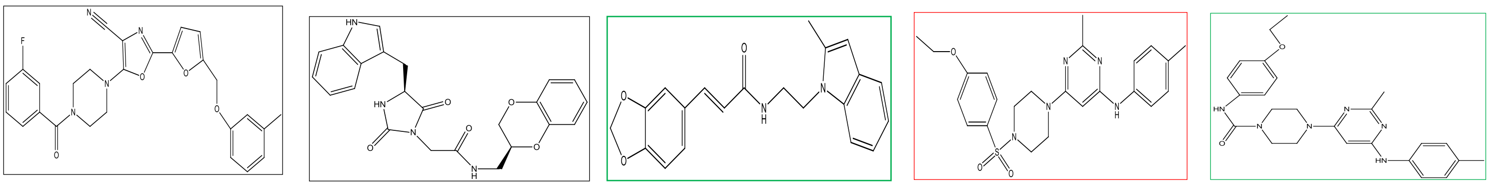

VNI-21 ZINC33011624 | VNI-22 ZINC09833912 | VNI-23 ZINC21794711 | VNI-24 ZINC21794721 | VNI-25 ZINC21795360

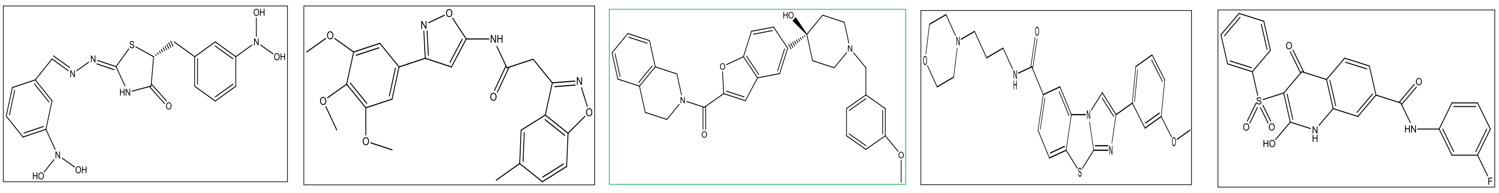

VNI-26 ZINC13476739 | VNI-27 ZINC09405560 | VNI-28 ZINC12149794 | VNI-29 ZINC20490345 | VNI-30 ZINC20530008

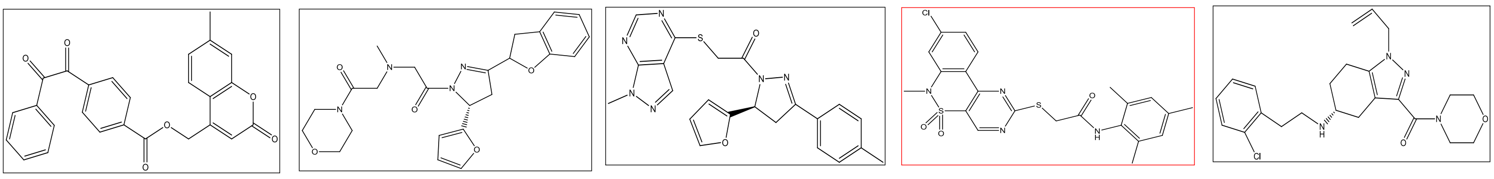

VNI-31 ZINC13146183 | VNI-32 ZINC12797304 | VNI-33 ZINC11681718 | VNI-34 ZINC33039637 | VNI-35 ZINC14716840

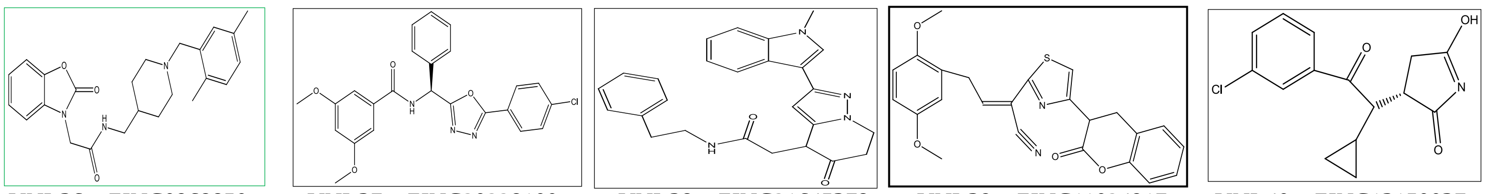

VNI-36 ZINC0968850 | VNI-37 ZINC20228100 | VNI-38 ZINC21645378 | VNI-39 ZINC11024917 | VNI-40 ZINC13150037

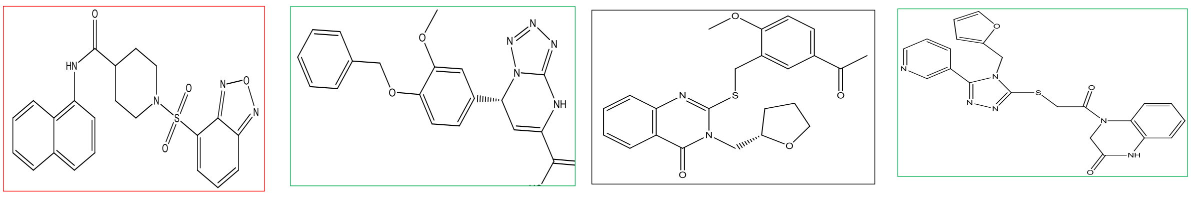

VNI-41 ZINC21157214 | VNI-42 ZINC15867266 | VNI-43 ZINC09472913 | VNI-44 ZINC13150037

Note that hits with 25% inhibition or more are colored in green and hits with 50% inhibition or more are colored in red.

Table C. Physical properties of the three active compounds

	LogP	H-bond donors	H-bond acceptors	Net charge	tPSA (Å ²)	Molecular weight	Rotatable bonds	$LogS_0$	Apolar desolvation (kcal/M)	Polar desolvation (kcal/M)
VNI-24	3.89	2	8	1	89	468.603	7	-4.21	9.08	-40.48
VNI-34	4.02	1	7	0	92	489.022	4	-4.38	8.44	-16.34
VNI-41	3.72	1	8	0	105	436.493	4	-3.77	7.56	-23.46

Log P, the lipo-hydro partition coefficient; S_0, the molar solubility (mol/L); tPSA, total polar surface area; logS0 =1.17 - 1.38*logP (Faller B, Ertl P (2007) Computational approaches to determine drug solubility. *Adv Drug Deliv Rev* 59: 533-545).

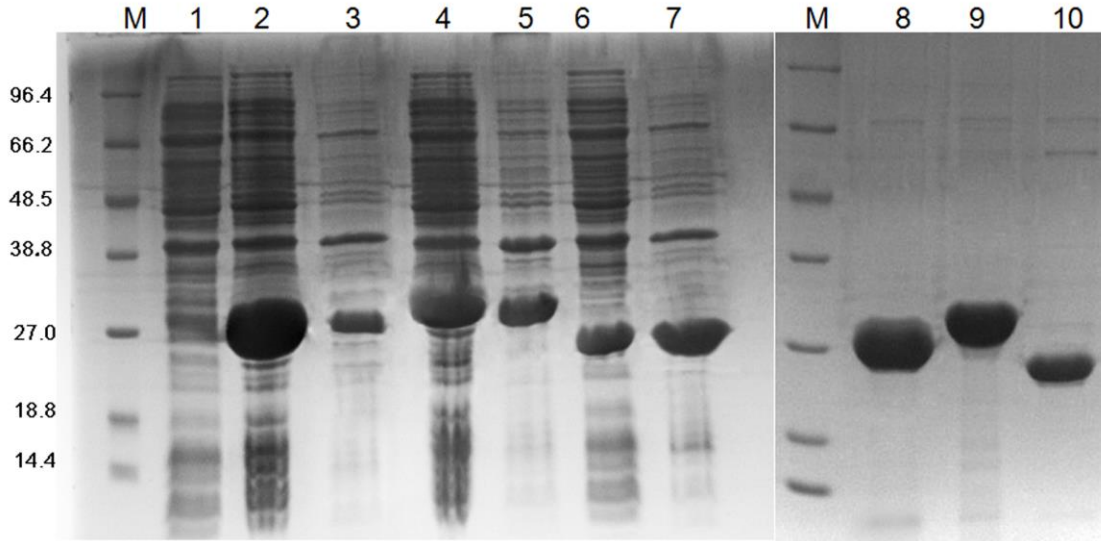

Figure A. Expression and purification of recombinant NDM-1, VIM-2 and SIM-1. Soluble, insoluble and purified fractions of the three recombinant MBLs was analyzed by SDS-PAGE. Lane M, molecular marks; lane 1, crude extract from *E. coli* with pET28 empty vector; lane 2, 4 and 6 supernatants of crude extract from *E. coli* with pET28-NDM-1, pET28-VIM-2 and pET28-SIM-1 respectively; lane 3, 5 and 7 precipitate of crude extract from *E. coli* with pET28-NDM-1, pET28-VIM-2 and pET28-SIM-1 respectively; Lane 7, 8 and 9 Nickel-affinity chromatography purified protein of NDM-1, VIM-2 and SIM-3 from the soluble part respectively.

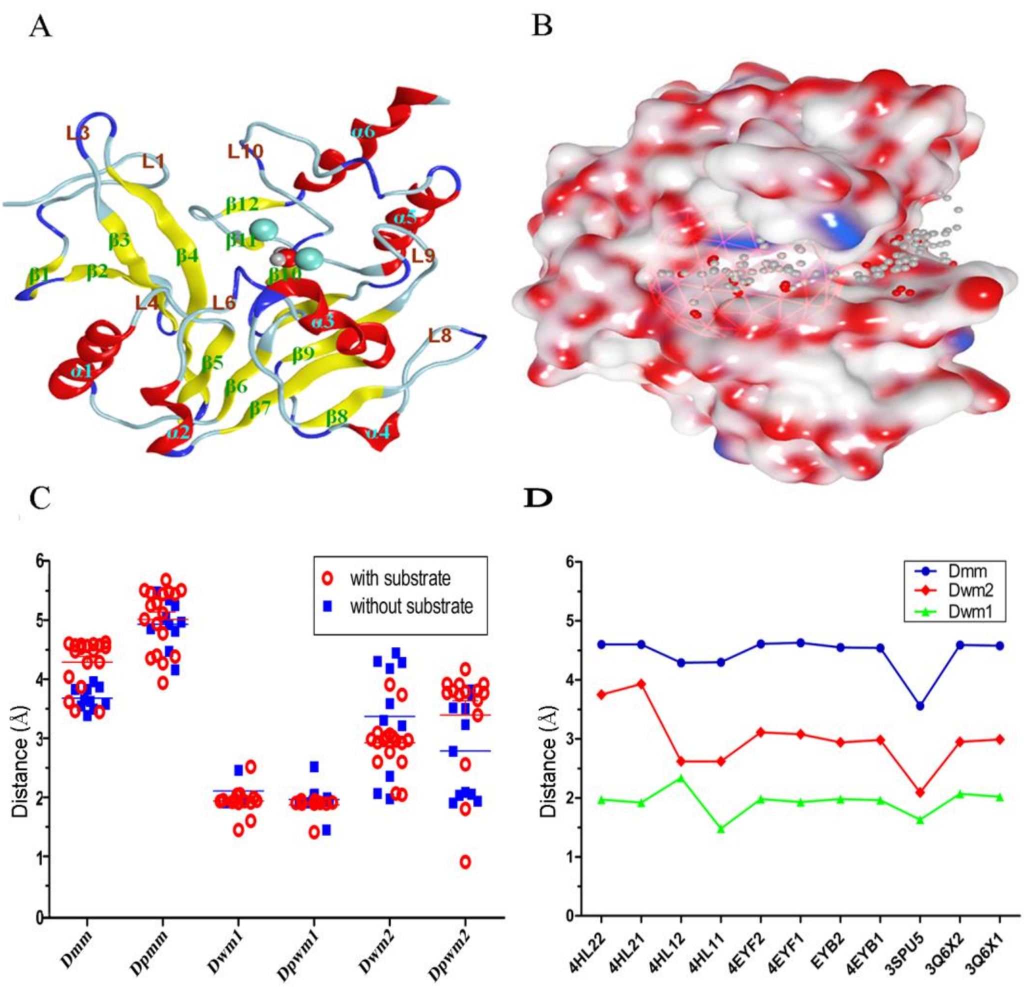

Figure B. The features of online reported NDM-1 crystal structures. (A) Ribbon diagram of NDM-1 structure (3Q6X: Loops, cyan; turns, blue; α-Helix, red; β-strands, yellow). (B) Molecular surface of NDM-1 structure represented as an Active LP image (green, hydrophobic surface regions; blue, mildly polarregions; and purple, hydrogen-bonding regions). Possible docking site around the active site calculated by Site Finder is rendered in alpha spheres. The docking box is restricted around the red mesh ball. (C) The distances between the two metal ions and between the metal ion with the bridging water in the reported structures and in the structures after optimization. Dmm: distances of metals in the active site of reported structures; Dpmm: distances of metals after energy minimization; Dwm1and Dwm2 referto the distances between the bridging water and Zn1 and Zn2, respectively while Dpwm1, Dpwm2 refers to the distance after energy minimization. (D) Dmm, Dwm1, Dwm2 was analyzed by Pearson correlation analysis. 11 chains of 6 PDB structures were retained when Dwm1 used a cutoff of less than 3 Å. The Pearson’s correlations for Dmm, Dwm2; Dmm, Dwm1; and Dwm1, Dwm2 are 0.747, 0.456, and 0.268, respectively.

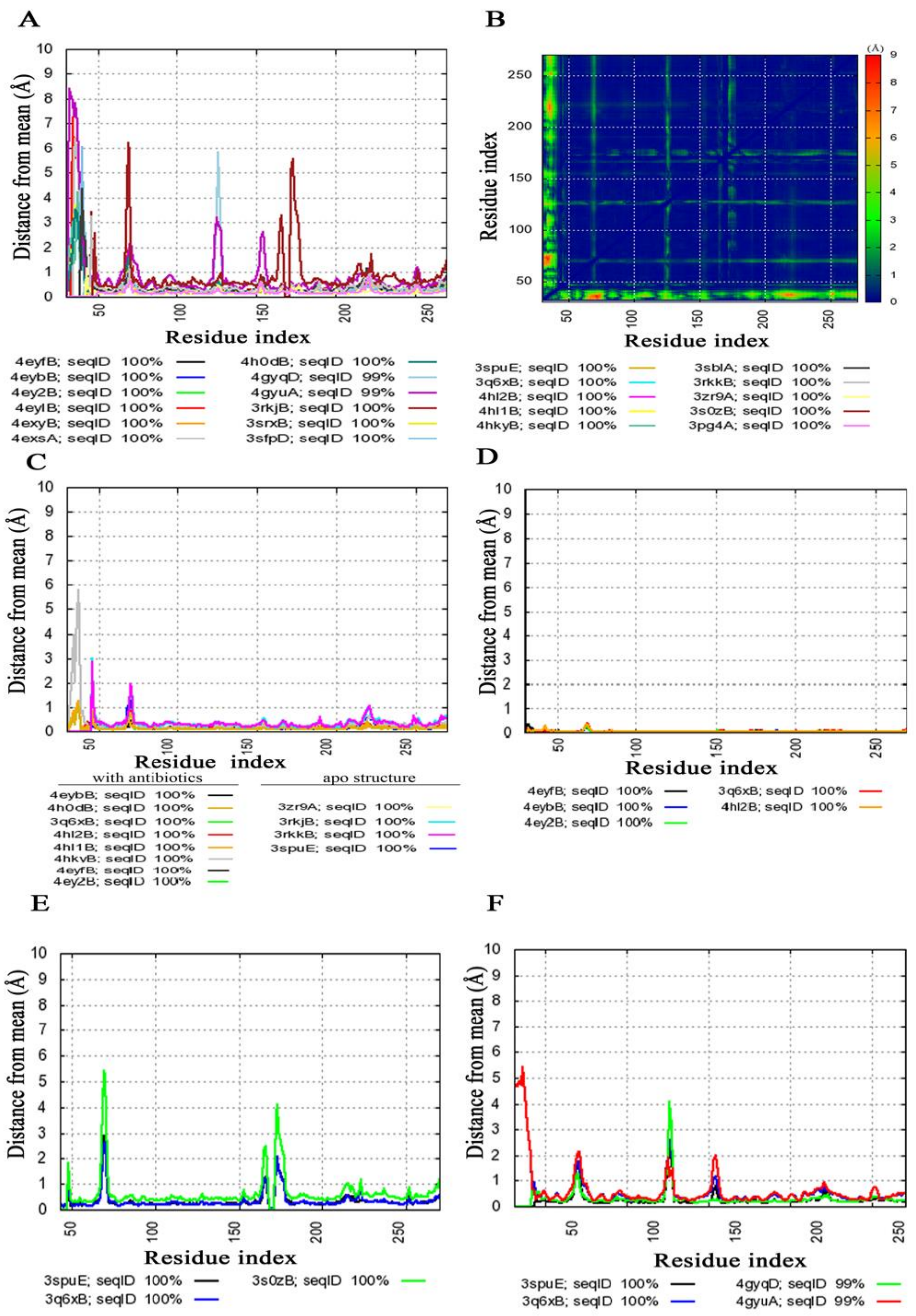

Figure C. Detail structural variability analysis of NDM-1 structures based on the PMP entry site. (A) Local (per residue) deviation of individual NDM-1 structures from mean of all structures based on a distance RMSD (dRMSD). (B) The variability matrix S (based on a weighted dRMSD) among NDM-1 structures (blue=low, green=medium, red=high). (C) NDM-1 structures with hydrolyzed substrates compared with apo NDM-1 structures. (D) Discrepancy between NDM-1 structures with different hydrolyzed antibiotics. (E) 3Q6X compared with NDM-1 mutants structures. (F) 3Q6X compared with 3S0Z and apo structure.

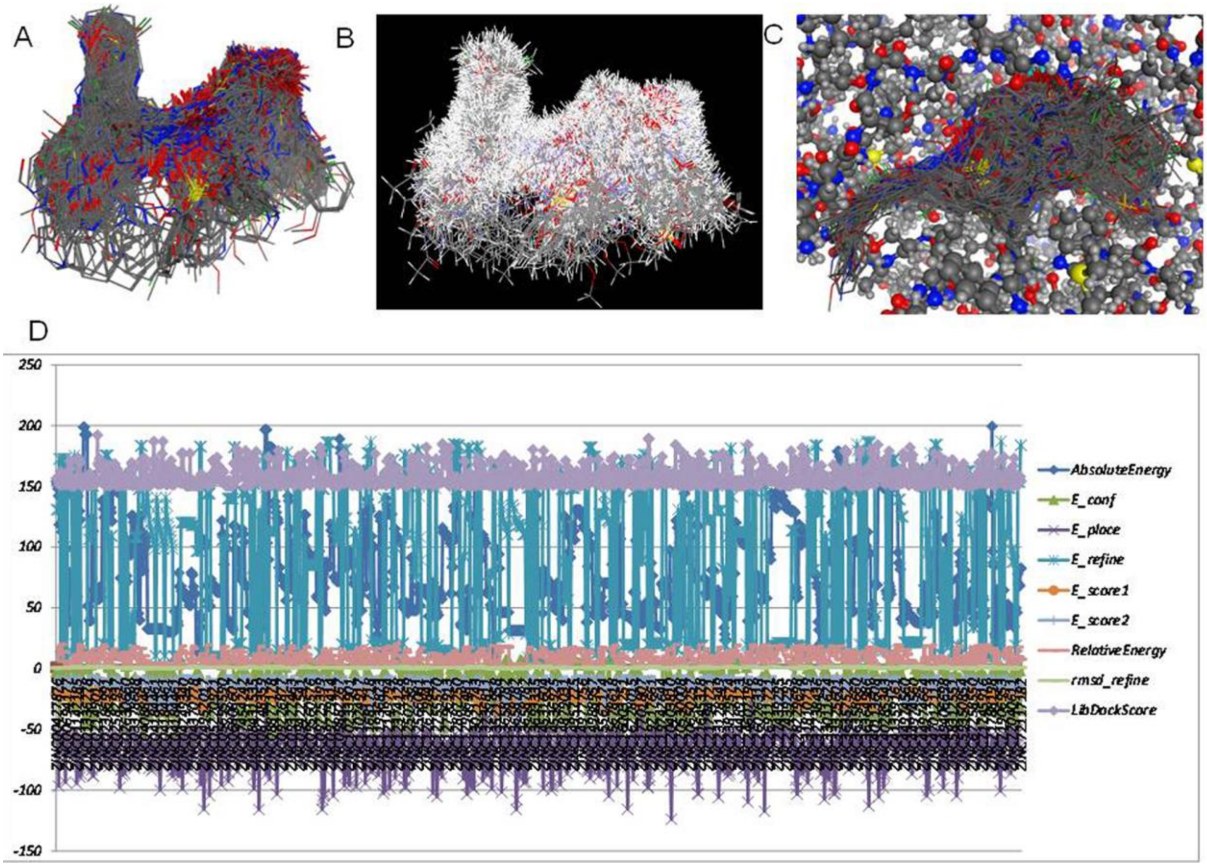

Figure D. Virtual screening of NDM-1 inhibitors. The conformations of 298 selected compounds produced by multistep docking process with (A) or without (B) hydrogen and the active site of NDM-1 (C). (D) The satisfied cutoff of different scores used in the multistep screening process: Absolute Energy under 200 kcal/mol; Relative Energy under 25 kcal/mol; LibDock Score above 150; E_conf under 9.00 kcal/mol; E_place under -17 kcal/mol; E_refine under 190 kcal/mol; E_score1 under -15 kcal/mol; E_score2 under -9 kcal/mol; rmsd_refie under 1.5 Å.

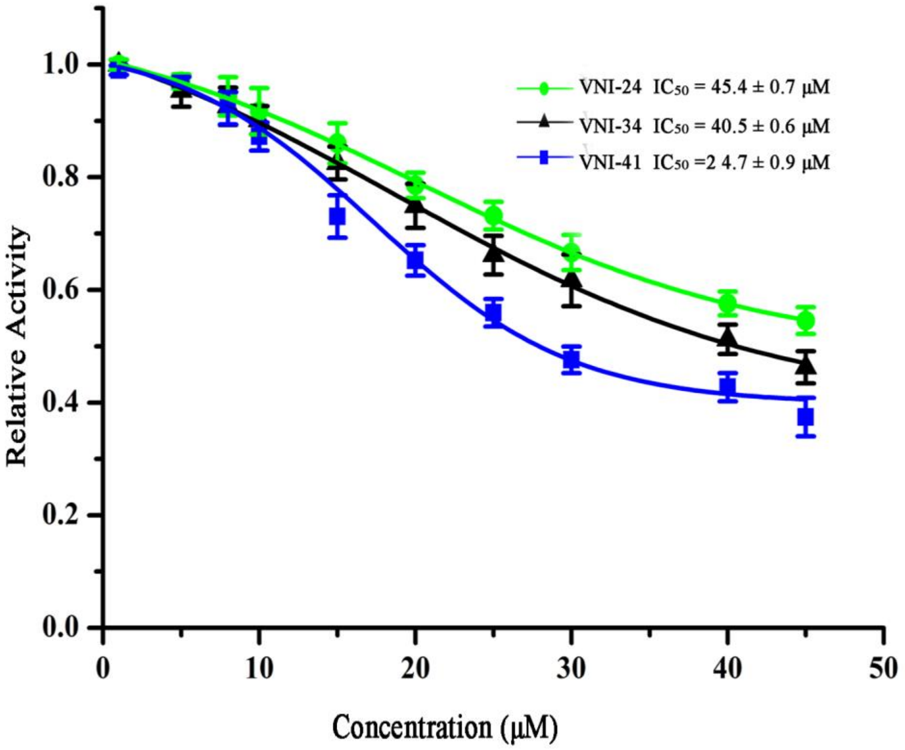

Figure E. Dose-dependent inhibitions of NDM-1 by VNI-24, VNI-34 and VNI-41 against NDM-1 using buffer containing 0.01% Triton X-100. Each data point indicated the remaining activity of NDM-1 after incubated with inhibitors, and were presented as mean ± standard deviation (n=4).
